# Modeling strategies for *in vivo* transcription factor binding predictions

**DOI:** 10.1093/bioadv/vbag123

**Published:** 2026-05-05

**Authors:** Ekin Deniz Aksu, Martin Vingron

**Affiliations:** Department of Computational Molecular Biology, Max Planck Institute for Molecular Genetics, Berlin 14195, Germany; Department of Computational Molecular Biology, Max Planck Institute for Molecular Genetics, Berlin 14195, Germany

## Abstract

Identification of *in vivo* transcription factor (TF) binding sites is crucial to understand gene regulation, but the lack of scalability in their experimental identification directs researchers towards computational models. These models are often specific for a given TF, which hinders their generalizability to held-out TFs. In this work, we analyse different modeling strategies to predict *in vivo* TF binding sites using DNA accessibility, TF RNA expression and binding motif features. We present and test a cross-TF transfer learning scheme that allows learning from the entire training set. We show that model ensembling and DNA language model embeddings increase model performance. We provide an analysis of feature importance and show that ground truth ChIP-seq data quality is an important determinant of model performance. We also test our models in an independent dataset of held-out TFs, and report a mean AUPR of 0.36 in a very challenging cross-TF, cross-cell-type and cross-chromosomal setting, providing estimates of binding for TFs without available ChIP-seq experiments.

## 1 Introduction

Gene expression is regulated by a set of transcription factors (TFs) that bind to regulatory regions on the DNA (Sa *et al*. 2018). Although sequence-specific binding preferences are known for many TFs, only a subset of predicted TF binding sites (TFBS) are occupied in any given cellular context—referred to as *in vivo* TFBS. Accurate identification of *in vivo* TFBS requires chromatin immunoprecipitation assays such as ChIP-seq (Johnson *et al*. 2007), which is impractical to perform across all 1600 human TFs on hundreds of cell types, due to their dependency on specific antibodies and the lack of parallelization or multiplexing.

Computational methods have emerged as powerful alternatives for predicting *in vivo* TFBS without the need for exhaustive experimentation. These methods broadly fall into three categories: (i) supervised tools that use motif position weight matrices (PWMs) along with DNase-seq, ATAC-seq and/or histone modification data, such as Virtual ChIP-seq, FactorNet, maxATAC and others (Pique-Regi *et al*. 2011; Chen *et al*. 2017; Qin and Feng 2017; Behjati Ardakani *et al*. 2019; Keilwagen *et al*. 2019; Li *et al*. 2019; Quang and Xie 2019; Fu *et al*. 2020; Karimzadeh and Hoffman 2022; Cazares *et al*. 2023). These tools are trained on TF ChIP-seq data and can make whole-genome TF binding predictions on unseen tissues from the provided data. (ii) Tools that are based on PWM-scanning in accessible regions, such as TF footprinting [HINT-ATAC (Li *et al*. 2019), TOBIAS (Bentsen *et al*. 2020)] or gene regulatory network (GRN) identification methods such as SCENIC+, Inferelator, GRaNIE, and most recently LINGER (Aibar *et al*. 2017; Skok Gibbs *et al*. 2022; Bravo González-Blas *et al*. 2023; Kamal *et al*. 2023; Yuan and Duren 2025). These methods are not trained on ChIP-seq data, and they usually predict TF binding in gene promoters, enhancers or ATAC-seq peaks as part of GRN analysis. (iii) Sequence-to-activity deep learning models such as DeepBind, Enformer and others (Alipanahi *et al*. 2015; Avsec *et al*. 2021a, 2021b; Wang *et al*. 2024). These methods learn the “complex grammar” of TF cooperation, but since they take only DNA sequence as input, they are limited to predictions on cell types in the training set.

Training supervised models requires high-quality ChIP-seq labels for each TF, which are not readily available for all TFs and cell types. Moreover, prediction accuracy is improved when data from multiple cell types are incorporated (Cazares *et al*. 2023). Therefore, efficient integration of multiple data sources and building prediction models that are generalizable to unseen TFs remain key objectives. Here, we test the impact of cross-TF transfer learning, incorporation of DNA language model embeddings and model ensembling on the TFBS binding prediction task. Transfer learning enhances prediction accuracy by using models that are pre-trained on other datasets, thus offering a way to integrate data efficiently. On the other hand, DNA language models such as DNABERT (Ji *et al*. 2021) and the Nucleotide Transformer (Dalla-Torre *et al*. 2025) extract high-level abstract features from DNA sequences, and can potentially improve generalizability if they learn universal rules regarding genomes.

Importantly, since TF binding predictions are TF- and cell type-specific, a unified prediction model would need to incorporate both elements in the input data for predictions in previously unseen TF and cell contexts. In practice, what is usually done is to either limit the model to predictions on the training set by a multi-task learning approach [e.g. Enformer (Avsec *et al*. 2021a)], or to give only the cell type-specific information such as DNA accessibility in the input data and train TF-specific models [e.g. FactorNet (Quang and Xie 2019)]. What we aim to achieve is a TF-agnostic model which is able to make predictions on any cell type for any TF including held-out TFs, which is quite challenging.

In order to assess different modeling strategies for cross-cell type and cross-TF evaluation scenarios, we train models that predict *in vivo* TFBS in cis-regulatory elements (CREs) using DNA accessibility, TF RNA expression and TF binding motif information. Since we aim to build TF and cell type-agnostic models, the input information should cover both aspects: DNA accessibility covers the need for cell type specific information, while TF binding motifs cover TF-related information. The usage of TF RNA expression is then necessary to distinguish TFs with similar motifs. We extract a set of handcrafted, biologically informed features from DNA regions and supplement them with embeddings from DNA language models. Using these features, we trained gradient-boosted decision tree models on ground-truth TF ChIP-seq data from the ENCODE-DREAM challenge. Our main goal is not so much to create a new state-of-the-art prediction tool, but to characterize feature importance, trade-offs of different modeling strategies and the determinants of performance.

For this end, we employed a novel two-stage transfer learning method that leverages available data across all TFs. First we built a general prediction model which is trained on all training TFs and cell types. Secondly, we train fine-tuned models for each individual TF, while incorporating the general model predictions as an additional feature. This way, we improve performance by using the knowledge gained from the entire dataset. Additionally, we explore feature importances and the factors driving variation in prediction performance across different TFs, and show that TFs with tissue-specific expression profiles are predicted more accurately than constitutively expressed TFs.

Finally, we test the performance of our models in previously unseen TFs, in a very challenging cross-TF, cross-tissue, and cross-chromosome setting. We show that our general model outperforms the mean TF-specific model ensemble in cross-TF predictions. Remarkably, this model maintains a strong predictive power even under these challenging conditions, allowing binding predictions for any TF with a known binding motif.

## 2 Results

### 2.1 Model overview and training scheme

The task is to predict TF binding probability in regulatory regions given a TF and a cell type. To achieve this, we incorporate handcrafted features that fall into four categories: (i) genomic features which depend only on the genomic region, (ii) motif features which depend on the region and the TF in question, (iii) cell type features which vary based on the region and the cell type, and (iv) cell-and-TF features which depend on both the TF and the cell type. A detailed description of all features and their definitions is provided in Sect. 4.

**Figure 1:**
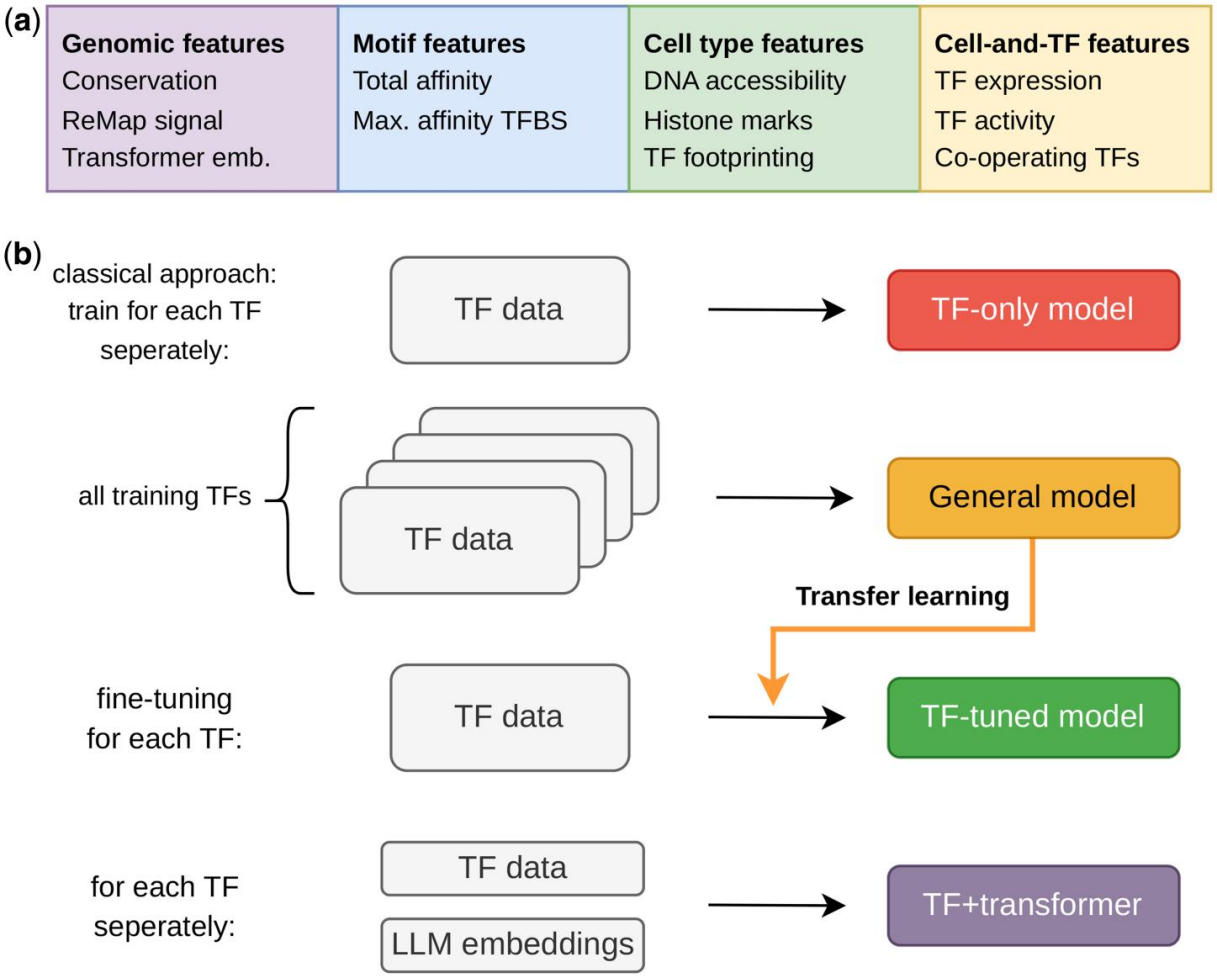
Schematic of the model training process. (a) Hand-crafted features can be divided into four categories: genomic features, motif-based features, cell type features, and cell-and-TF features. (b) Our model training setup. TF-only models only use data from one TF. The general model is trained on all training TFs in a TF-agnostic framework. Afterwards, this model is fine-tuned on each TF separately, by training models on each TF, after incorporating general model prediction as an additional fea-ture (TF-tuned). TF+transformer models additionally use genomic LM embeddings.

We developed four different types of gradient-boosted decision tree models. First, a “general model” was built, which pools all available training data across TFs and cell types, allowing the model to generalize across different conditions. Each genomic region is included multiple times in the training set, as many as the number of different TF-cell type pairs. This significantly expands the training set which is beneficial for learning optimal parameters. Second, we developed the so-called “TF-tuned” models, which are trained from scratch only on the data from a single TF, allowing the model to be tuned specifically for this TF. Importantly, to leverage the knowledge benefits of the entire dataset, these models have the “prediction by the general model” as an additional input feature, which can be seen as transfer learning across TFs. Third, we developed “TF-only” models which only use training data from the selected TF, using the same feature set as the general model. For TF-tuned and TF-only, we also trained “criss-cross” ensemble models, where each model fold is trained on a different set of cell types and chromosomes, while being validated on the rest. Lastly, our ensemble “TF+transformer models” use, in addition to the handcrafted features, 1024-dimensional embeddings from the DNA language model Nucleotide Transformer (Dalla-Torre *et al*. 2025) (Fig. 1).

Our training data comes from three main sources: ATAC-seq and RNA-seq data from the ENCODE project (Moore *et al*. 2020) and TF binding motifs from the TRANSFAC (Matys *et al*. 2006) database. We further utilize precomputed (López Ruiz de Vargas 2021) enhancer probabilities derived from histone modification ChIP-seq data using the CRUP tool (Ramisch *et al*. 2019). Training genome regions were defined as all ENCODE candidate cis-regulatory elements (cCREs) (Moore *et al*. 2020), excluding regions on chromosomes 1, 8, and 20, which were reserved for testing. Altogether, our training dataset contains 8 cell types, 29 TFs and 75 TF-cell type pairs.

### 2.2 Accurate prediction of *in vivo* TF binding sites

We evaluated the performance of our models on 36 TF-cell type pairs: 12 from the “final round” and 24 from the “leaderboard” dataset of the ENCODE-DREAM challenge. For each test region, we compared model predictions of TF binding to the ground truth TF ChIP-seq labels provided by the challenge.

Our evaluation metric is the area under the precision-recall curve (AUPR), chosen due to the significant class imbalance in the dataset, where bound labels represent only ∼2% of samples. In contrast, the area under the receiver-operating characteristic (ROC) curve fails to differentiate between strong and weak models (Fig. S1).

**Figure 2:**
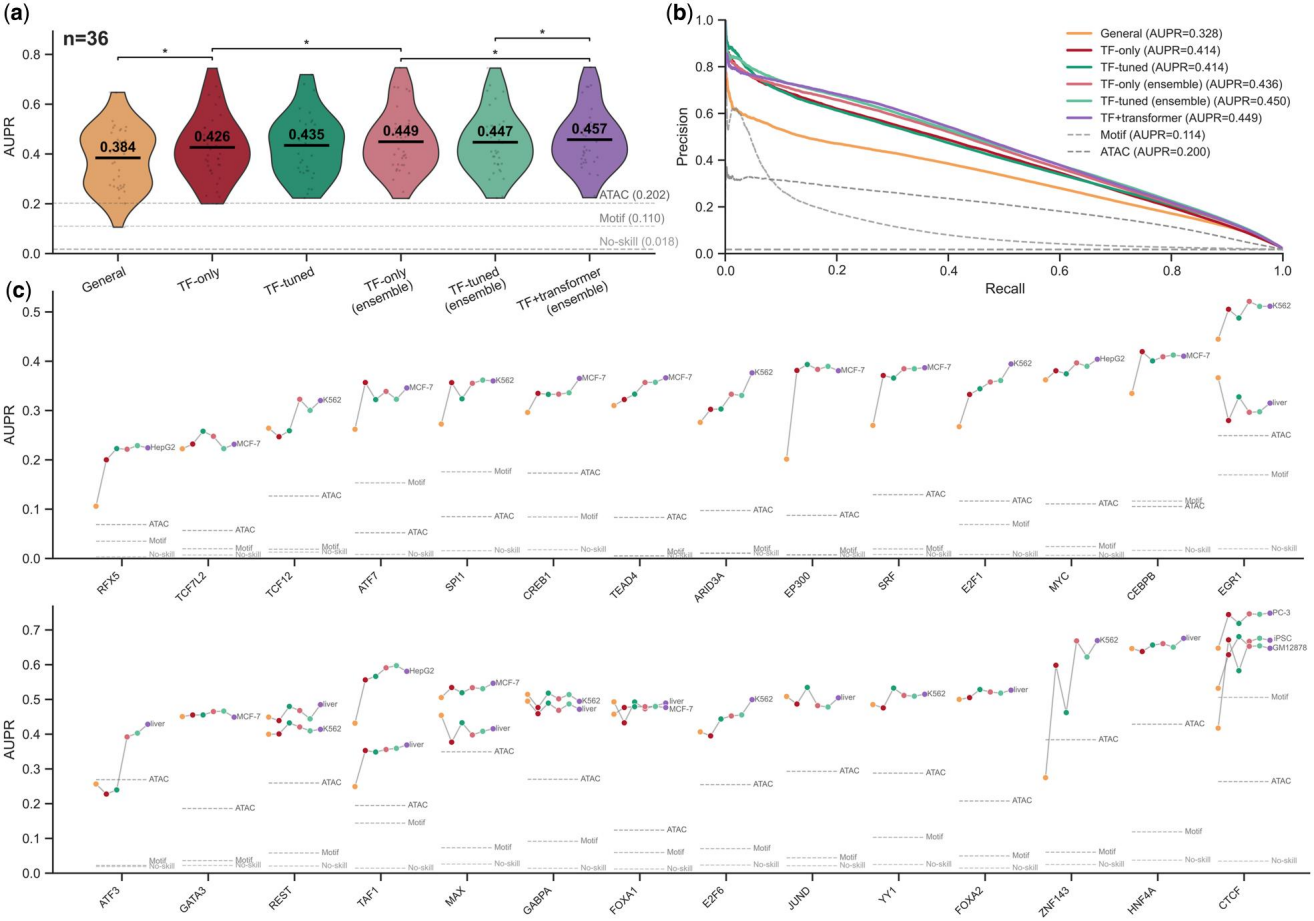
Accurate prediction of cell type specific *in vivo* TFBS. (on previous page). (a) Area under the precision-recall curve (AUPR) scores for 36 TF-tissue pairs from the final round and leaderboard datasets. Mean values are reported inside the violin plots. Selected significantly different distributions are marked with an asterisk (paired *t*-test, *P* < .05). Mean AUPRs with no skill, motif scanning (TRAP) and mean ATAC-seq signal baselines are designated with dashed lines. (b) shows the same data in a pooled PR curve, and (c) shows the same data across all TFs in the dataset. Each model’s AUPR is represented by a dot, and dots for the same cell type are connected. The dashed lines represent the aver-age AUPR of the baselines in each TF.

Our models show good overall performance with mean AUPR scores up to 0.457, compared to ATAC and motif scan baselines with 0.2 and 0.11 AUPR, respectively (Fig. 2a). Mean AUPR for random guessing is 0.02, which corresponds to the ratio of TF-bound enhancers in the test set. The TF-agnostic general model can be improved with transfer learning (TF-tuned 0.435 vs General 0.384), however this yields marginal increases over TF-only models (0.426). Criss-cross ensembling of models also improves performance (e.g. TF-only ensemble 0.449 vs TF-only 0.426). Finally, the best model was the TF+transformer model, with a mean AUPR of 0.457. Using genomic language model (LM) embeddings improves prediction performance, however embeddings alone significantly underperform compared to the full model (Fig. S2). Looking at the pooled precision-recall (PR) curve, we see that models have similar behaviours with gradually declining PR curves (Fig. 2b). However, interestingly, the motif scan baseline has very high precision initially, which drops sharply with increasing recall. On the other hand, the ATAC-seq baseline has a smoother overall curve. So, motif scanning can identify sites with a very strong match to the known motif, but cannot pick up sites with lower affinity.

Performances of these models changed substantially based on the chosen TF (Fig. 2c). The highest score was obtained for CTCF, which is easier to predict than most TFs, probably due to its high motif information content and constitutive binding across different cell types. Interestingly, there were some TFs where performance deviated from the general trend. For example, for EGR1, GABPA, FOXA1 and MAX in liver, the general model had the best performance among all tested models. We hypothesized that the weakness of TF-specific models in these TFs may be due to lower data quality compared to others, allowing the general model to leverage the information from other, higher-quality datasets. To test this hypothesis, we investigated three commonly used ENCODE data quality metrics: fraction of reads in peaks (FRiP) and PCR bottlenecking coefficients 1 and 2 (PBC1 and PBC2). Indeed, we found that these four datasets have significantly lower FRiP scores than other datasets (mean 0.03 vs 0.08, *t*-test *P* < .01). Interestingly, we also found that FRiP and AUPR scores from the TF+transformer model are correlated (Spearman’s rho = 0.66, *P* < .0001). This underlines ground truth data quality as one of the important determinants of predictive power (Fig. S3a).

**Figure 3:**
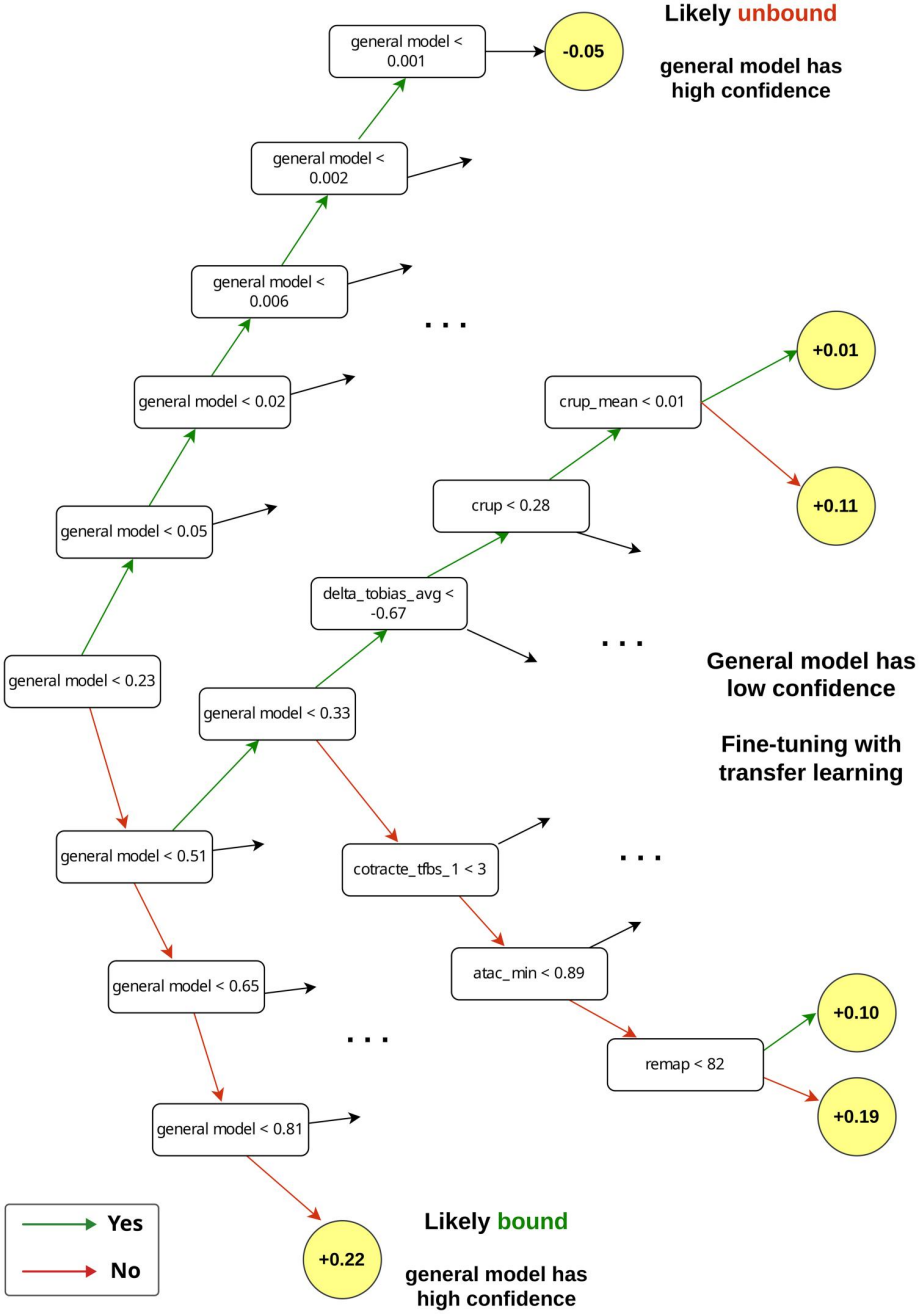
Transfer learning-informed decision trees. An excerpt from a representative decision tree from the FOXA2 TF-tuned model. Each node shows a question such as “Is the general model score less than 0.23?”, and green arrows indicate a positive answer while red arrows indicate a negative answer. Nodes with a yellow background correspond to output leaves indicating predictions. Top and bottom paths denote paths where the general model has high confidence, and hence the model does not probe other features. In the middle of the tree, the general model score is intermediate, and fine-tuning is achieved by probing other features in a TF-specific manner.

Close examination of a single decision tree from the TF-tuned FOXA2 model reveals transfer learning in effect. The tree initially checks the value of the general model score. If this score is close to zero, no additional features are queried and the prediction is steered towards an unbound state. Conversely, a very high prediction score pushes the prediction towards a bound state. These represent cases where the general model has high confidence. In contrast, if the general model has low confidence, represented by an intermediate score, the tree proceeds to evaluate additional features such as enhancer activity or co-operating TFBS, thus further refining its prediction (Fig. 3).

We have selected three top-performing methods from the ENCODE-DREAM challenge for comparison to external tools: autosome.ru (https://www.synapse.org/Synapse: syn8006653/), FactorNet (Quang and Xie 2019) and J-Team (Keilwagen *et al*. 2019), with the latter two tied for first place. Comparisons with these methods act as a sanity check relative to the historical state-of-the art performance on the same ChIP-seq training set. Our TF+transformer model’s mean AUPR of 0.505 was the highest, followed by J-Team (0.48), FactorNet (0.46) and autosome.ru (0.46) (Fig. S5). Remarkably, even our general model performed comparably to autosome.ru and FactorNet, while the TF-tuned and TF+transformer models were further ahead.

It is important to highlight that models from the DREAM challenge had a much larger context window spanning multiple kilobases, which is advantageous for performance. In contrast, our models were deliberately designed with a constrained context window of one enhancer (max. 350 bp), prioritizing interpretability over performance. On the other hand, our predictions use ATAC-seq data while the original DREAM challenge data is DNase-seq. A more detailed examination of method comparability is provided in the Supplementary Information.

### 2.3 Analysis of feature importance

We systematically tested the contribution of different features to model performance by omitting specific feature sets from the training set and retraining general and TF-tuned models. Since many of our features are interdependent, we omitted subsets of features as opposed to single features. For example, motif-based features were excluded together as they are highly correlated.

**Figure 4:**
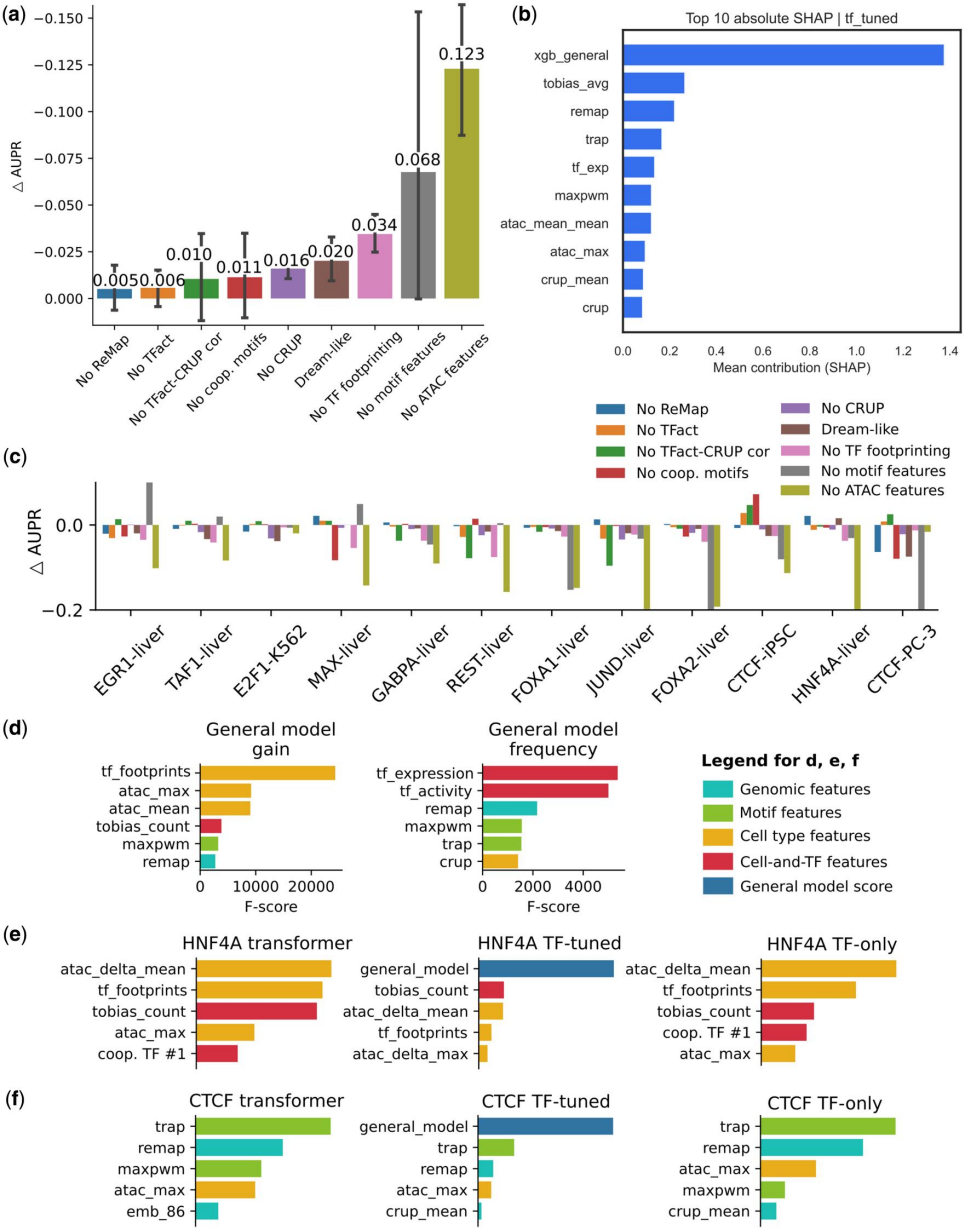
Feature importance. (a) The change in AUPR (ΔAUPR) compared to the full TF-tuned model after masking specific feature sets in the final round dataset. Error bars indicate variance across TF-cell pairs (*n* = 12). Note that the y-axis is flipped. (b) Top 10 mean absolute SHAP values across all trained TF-tuned models. (c) Breakdown of data from (a) across TF-cell pairs. (d) Feature importance ranking based on XGBoost F1-scores, with gain and frequency metrics. (e) Feature contributions in different HNF4A and (f) CTCF models. Features are colored according to their class.

On average, ATAC-seq-derived features were the most impactful, causing a notable AUPR drop of 0.12, which represented 24% of the base performance. Motif-based features followed with a decrease of 0.07 AUPR. In contrast, removing other features such as CRUP enhancer scores, TF activity or ReMap scores had lower impact (Figs. 4a and S6).

ATAC-seq features were the most important in 8 of the 12 TFs tested, consistently making significant contributions, with the exception of E2F1 in K562 cells and CTCF in PC-3 cells. On the other hand, motif-based features showed a more complex contribution pattern. For TFs such as EGR1, TAF1, HNF4A, and MAX, omitting these features slightly improved performance. TF activity-CRUP score correlations were important for GABPA, REST and JUND models, but they are detrimental to performance in CTCF (Fig. 4c). A closer look at individual features revealed that the TF footprinting score stands out as the single most influential feature in the general model (Fig. 4d). TF footprinting incorporates both accessibility and binding affinity, while accounting for local decreases in accessibility due to TF binding. Therefore, it captures a high degree of information regarding TF binding events. Also, when analyzing feature importance using XGBoost (Chen and Guestrin 2016), we found that it is important to choose the correct metric: the “frequency” metric is misleading because it simply counts how often a feature is included in decision tree splits, and less important features are sometimes included more often.

The importance of specific features also varied by TF and model architecture. For HNF4A, features such as ΔATAC, TF footprinting and bound TFBS counts are important in TF+transformer and TF-only models. In the TF-tuned model, expectedly, the general model score was by far the most important feature (Fig. 4e).

Interestingly, for the CTCF TF+transformer model, sequence-based features were in the lead, with total motif affinity being the most important. One of the DNA language model embedding dimensions was also in the list of top features. It is possible that this dimension captured some sequence feature regarding CTCF binding (Fig. 4f).

In addition to these ablation experiments, we also analysed feature importance in TF-tuned and other models with Shapley value approximations using the SHAP package (Lundberg and Lee 2017). Unsurprisingly, the general model prediction is the biggest contributor, followed by TF footprinting signal from TOBIAS, and ReMap score, TRAP motif scanning, and TF expression (Figs. 4b and S7).

SHAP analysis allows assessment of how feature contributions change with respect to feature values using dependence plots, which we analysed (Fig. S8). TF footprinting signal is a consistently important feature, with low values decreasing the prediction score and high values increasing it. Interestingly, ReMap scores are more informative when they are close to zero. With increasing ReMap scores, the increase in prediction score has diminishing returns. However, from the ablation analysis we know that omitting ReMap scores does not decrease performance dramatically. Combined, these observations suggest that our models are using ReMap signals as a “shortcut” to detect unlikely binding sites. If the mean ChIP-seq signal across all TFs and tissues/cell lines is zero, it is unlikely that this region will be bound by any TF. However this is likely detectable by a combination of other features. Also interestingly, we see that most models consider a TF expression of zero as an important negative factor. However, if the TF is expressed in the given tissue, then the expression level does not seem to matter, as the SHAP values fluctuate around zero. This is consistent with known biology, as many TFs have low mRNA expression levels compared to other genes, yet can fulfill their function.

### 2.4 Prediction performance on HNF4A in example locus

**Figure 5:**
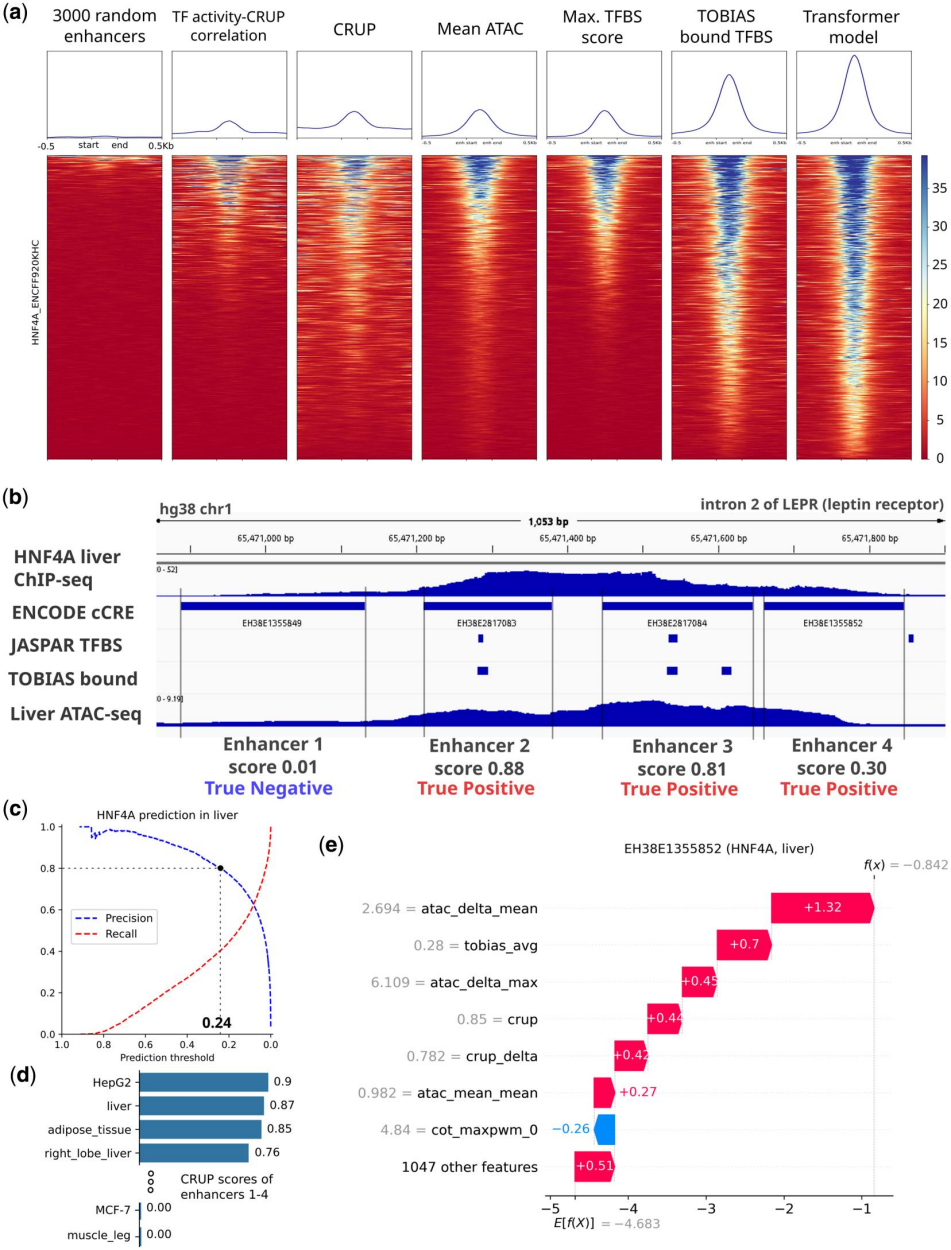
Epigenomic signals from selected enhancers. (a) HNF4A ChIP-seq signal (fold change compared to control) from liver cells, centered on enhancers ranked by various metrics. (b) ChIP-seq, ATAC-seq signals, and TFBS from four example enhancers located in intron 2 of the LEPR gene. (c) Precision and recall curves for HNF4A in liver tissue, (d) Mean CRUP enhancer scores for the selected enhancers. (e) SHAP feature contributions to the prediction score for Enhancer 4.

We analyzed the HNF4A ChIP-seq signal in liver tissue in different genomic regions, comparing 3000 randomly selected enhancers to the top 3000 enhancers ranked by various features (Fig. 5a). Regions ranked by the highest correlation between TF activity and CRUP scores show a signal in nearly half of the regions, centered around selected enhancers. The signal intensity increased when using CRUP scores or ATAC-seq signals. Ranking enhancers by TFBS score results in a clear and strong signal from the top-ranked enhancers but the signal drops off faster compared to ATAC-ordered regions. Using the number of TF footprinting-predicted bound TFBS gives a better result. The best result is achieved by the TF+transformer model, where a strong signal centered around the enhancer is present in almost all of the top 3000 predictions (Fig. 5a).

To assess prediction accuracy at a specific locus in the human genome, we examined four enhancers located in intron 2 of the *LEPR* (leptin receptor) gene, which is linked to feeding response and likely to be active in liver tissue. This locus is accessible in liver, with HNF4A binding sites present in enhancers 2 and 3. Our TF+transformer model predicts HNF4A binding probabilities of 0.01, 0.88, 0.81, and 0.3 for enhancers 1 through 4, respectively. Overlaying with the HNF4A ChIP-seq signal, the predictions align well with the observed binding pattern (Fig. 5b). Precision and recall analyses indicated that the threshold for 80% precision is around 0.24, confirming enhancers number 2, 3 and 4 as true positives, while enhancer 1 is a true negative (Fig. 5c).

Enhancer activity profiles of the selected enhancers show that top ranked biological states are relevant to liver, including HepG2 (hepatocellular carcinoma cell line), liver, adipose tissue and right lobe of liver, all showing high enhancer probabilities. In contrast, enhancer activity drops off sharply in other tissues, many with CRUP scores of zero. This pattern is consistent with the observation that these enhancers are likely liver-specific.

Local SHAP analysis allows us to understand which features influence model decisions for individual enhancers. For example, in Enhancer 4, the mean ATAC-seq signal was higher than the mean across other cell types, which led to the biggest increase in prediction score. Other important factors were high TF footprinting score and high CRUP enhancer score (Fig. 5e).

### 2.5 Prediction performance indicators

To explore potential predictors of model performance, we examined correlations between performance and TF-related indicators such as mRNA expression levels and TF activity. TF activity is defined as the contribution of each motif to cell type-specific enhancer activity (see Sect. 4). For each TF-tissue pair, we plotted model performance against TF expression in the relevant tissue. We excluded CTCF because its exceptionally constitutive binding makes it much easier to predict than other TFs. We observed no statistically significant linear relationship between model performance and TF expression. However, when TF activity was substituted for expression, a statistically significant relationship emerged (Pearson’s *P* < .05), indicating that TF activity is a better predictor of performance (Fig. S9a, b).

We hypothesized that the predictability of TF binding might differ between tissue-specific and broadly expressed TFs. Given the binding motif, DNA accessibility and TF expression; tissue-specific TFs could be on average more predictable, since constitutively expressed TFs with simple motifs generate an abundance of candidate sites which can confuse machine learning models. To test this hypothesis, we quantified tissue specificity using expression entropy, calculated across the expression profiles of TFs in 80 different conditions. Low entropy indicates tissue-specific expression, whereas high entropy reflects a more uniform, ubiquitous expression. Our results revealed a negative correlation between expression entropy and model performance (Pearson’s *R* = −0.51, *P* < .01), though the effect is mainly driven by a few outliers (Fig. S9c, d). It should be noted that CTCF is an exception to this trend, as it exhibits both constitutive binding and expression, and also has a very well-defined binding motif. These characteristics allow for highly accurate prediction of CTCF binding, despite its broad expression profile.

In order to account for experimental noise and the limitations of binary ChIP-seq binding labels, we also assessed performance using mean ChIP-seq signal (fold change over control). Ideally, the AUPR of using quantitative ChIP-seq signal to predict ChIP-seq labels should approach 1. However, due to the signal-noise ratio of the experiment, the technical noise from peak-calling and the fixed genomic coordinates, which may not perfectly align with ChIP-seq peaks, the actual performance is reduced.

Our analysis showed that AUPRs using ChIP-seq signal ranged from 0.76 to as low as 0.27 for some TFs. For example, GABPA in liver achieved an AUPR of only 0.27, whereas our TF+transformer model reached an AUPR of 0.47, exceeding the ChIP-seq performance by more than 70%. Another example is EGR1, where both ChIP-seq and the TF+transformer model yielded similar AUPRs of 0.34 and 0.31, respectively. Performances in different TFs can be normalized in this manner relative to the ChIP-seq prediction performance. This metric helps to highlight TFs where there is room for improvement. For instance, TCF7L2 exhibited the lowest relative performance, with a TF+transformer model AUPR of 0.23 compared to 0.64 for ChIP-seq, representing only 36% of the ChIP-seq level. On the other hand, despite similar absolute AUPR scores for EGR1 and ATF7 (0.31 and 0.34, respectively), their relative performances diverged: EGR1 achieved 92% of the ChIP-seq performance, while ATF7 only reached 50%. This suggests that improving ATF7 predictions may be easier than improving EGR1 predictions, as the latter is already nearing the upper bound imposed by ChIP-seq performance (Fig. S9e).

### 2.6 Predicting previously unseen TFs

To systematically evaluate the performance of our models on previously-unseen TFs, we curated a new ChIP-seq dataset containing NR2F2, ZBTB33, RXRA, RAD21, and HNF4G in liver tissue and ISL1, TFAP2B, FOSL2, NFIC, and PBX3 in the SK-N-SH neuroblastoma cell line. The only TF-related information the models have access to are their binding motif PWMs and their mRNA expression. The predictions we make on these TFs are simultaneously cross-TF, cross-cell type and cross-chromosome, representing the most challenging prediction scenario.

**Figure 6:**
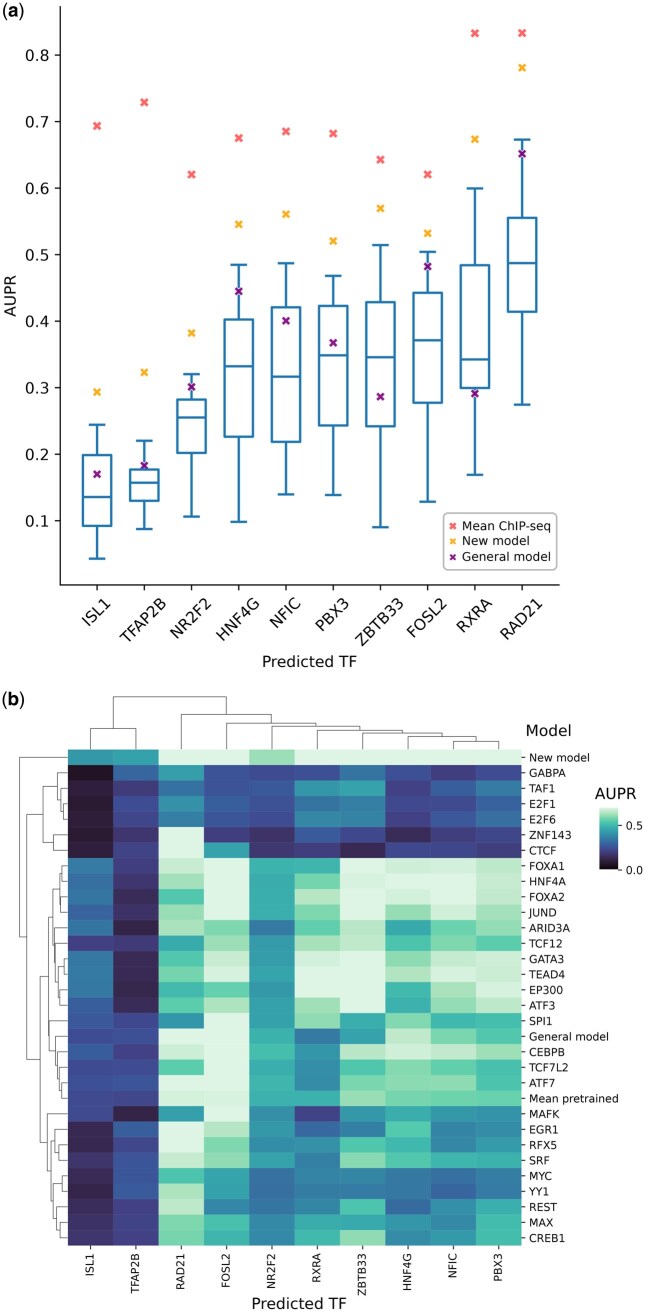
Predicting binding of previously unseen TFs. (a) Prediction performance for 10 newly introduced TFs using pre-trained TF+transformer models (*n* = 29 for each new TF). Yellow crosses denote the performance of models trained on data from the new TF, purple crosses denote the performance of the general model, and red crosses represent the performance based on average ChIP-seq signal. (b) Heatmap and dendrogram showing the prediction performance of each predicted TF-model TF pair.

We predicted the binding of each new TF using four different approaches: (i) each of the previously trained TF+transformer models, (ii) the general model, (iii) a newly-trained TF+transformer model trained on the new TF data, and (iv) mean ChIP-seq fold change signal (Fig. 6a). The mean performance of our TF+transformer models lies between 0.15 (predicting ISL1) and 0.48 (RAD21). Unsurprisingly, newly trained TF+transformer models outperformed pre-trained models in all cases, and using the mean ChIP-seq signal was the best predictor.

We observed that some of the TF models are good predictors of a new TF, while ineffective at predicting others. For example, when predicting ZBTB33 binding, the GATA3 model was the best performing TF model, having an AUPR of 0.51—nearly matching the performance of the newly trained ZBTB33 model (AUPR 0.57). However, the GATA3 model is one of the worst predictors when predicting TFAP2B. Conversely, GABPA is the best TF model for TFAP2B, but it has poor performance in ZBTB33 (Fig. 6b). We were unable to determine a consistent rule for selecting the best TF-specific model for a newly introduced TF. Neither the similarity of expression profiles of the two TFs, nor the similarity in their binding motifs have a correlation with model performance, the latter being consistent with previous reports (Liu *et al*. 2017).

Analyzing all combinations of model TFs and predicted TFs revealed distinct patterns. ISL1 and TFAP2B are challenging to predict, regardless of the model used. Furthermore, models fine-tuned on GABPA, TAF1, E2F1, E2F6, ZNF143 and CTCF consistently underperformed when predicting previously unseen TFs. Interestingly, the CTCF model can make good predictions only on RAD21, a member of the cohesin complex which cooperates with CTCF to regulate chromosome folding (Mach *et al*. 2022) (Fig. 6b).

Remarkably, the general model demonstrated robust generalization to previously-unseen TFs, achieving up to 0.65 AUPR (mean 0.36 across 10 new TFs) and outperforming the median performance of the TF+transformer models in 8 out of 10 TFs (Fig. 6a). Moreover, the general model also surpassed the ensemble mean prediction of the previously trained models.

### 2.7 ChIP-seq data quality is a determinant of model performance

We tested the relationship between mean ChIP-seq AUPR and the three quality control metrics described above (FRiP, PBC1, PBC2) on the new TFs. Unsurprisingly, we found a significant correlation between FRiP and AUPR (Spearman’s rho = 0.79, *P* < .01, Fig. S3b). PBC scores were not significantly correlated with performance.

Performance of the general model on the 10 new TFs is not correlated with FRiP scores (*P* = .8, Fig. S3c). However, FRiP scores of these TFs are all higher than 0.025. To evaluate the impact of even lower FRiP, we introduced 9 new TFs with low FRiP into the dataset. Interestingly, both general model AUPR and mean ChIP-seq AUPR are significantly correlated with FRiP in the expanded dataset (*P* < .001, Fig. S4). A closer look at FRiP scores suggests that scores less than 0.02 (less than 2% of reads in called peaks) is associated with a mean ChIP-seq AUPR of less than 0.35 and general model AUPR of less than 0.15. After the threshold of 0.02 FRiP, mean ChIP-seq AUPR jumps to around minimum 0.6.

Based on this analysis, we suggest that ChIP-seq datasets with less than 0.02 FRiP should be discarded, as their signal-to-noise ratio does neither allow for accurate ground truth labels nor for training accurate models.

## 3 Discussion

Our study highlights four main findings: (i) we introduce a novel two-stage transfer learning training approach that leverages the full training set while fine-tuning models for specific tasks, (ii) using DNA language model embeddings as independent features enhances the performance of simpler models, (iii) TF-agnostic models can be generalized to make cross-TF, cross-cell type and cross-chromosomal binding prediction, and (iv) accurate models require high quality ground truth ChIP-seq labels with higher than 0.02 FRiP.

Training prediction models that can generalize across different TFs and cell types is a long-standing goal in computational biology. On paper, such models enjoy an advantage in bigger training sets, the potential to yield deeper biological insights and of course, being able to make predictions on previously-unseen samples. However, despite the potential appeal, this approach is not commonly used in previous work. Instead, most prediction models use data from a single TF at a time. Recently, transfer learning was used to improve performance of a convolutional neural network to predict TF binding sites (Novakovsky *et al*. 2021). However, unlike the previous approach, our method can make predictions in cell types that were not part of the training set, which is crucial for real-world applications.

Features that are most important for TF binding prediction are DNA accessibility and sequence affinity to TF binding motifs. Other features such as enhancer probability score, TF activity, ReMap score or conservation score all contribute slightly. The single best analysis for TF binding prediction we find to be TF footprinting analysis. This is not surprising because it is in essence a hybrid feature very close to the underlying biology, since it combines accessibility information with TF binding site information, while also taking the shape of ATAC-seq coverage into account for increased accuracy.

Furthermore, our results show that the general TF binding prediction model makes reasonably accurate predictions in previously-unseen TFs, outperforming the ensemble of pre-trained TF-specific models. We extend the findings of Liu *et al*. (2017) on model generalization, by incorporating more powerful and accurate learning algorithms and analyzing model selection strategies. Moreover, we enhanced predictive performance by integrating sequence embeddings from a DNA language model as additional features, which significantly boosts the model’s capacity to capture complex sequence motifs.

In conclusion, computational prediction of TF binding has made significant progress in the recent decades and can provide increasingly reliable estimates of TF binding. With the growing availability of data, such as the ReMap database with binding data on 1200 TFs, transfer learning approaches present a powerful opportunity to leverage this vast source of information and develop highly accurate prediction models. Additionally, the integration of foundation models such as DNA language models enables capturing higher-level sequence features, which provide higher accuracies. With these developments, computational prediction performance is getting closer to the accuracy of ChIP-seq experiments, even though it is not yet fully capable of matching them. Looking ahead, accurate binding predictions for all TFs would be very beneficial for large scale screening applications and mapping gene regulatory networks. The development of a unified model of transcription factor binding with experiment-level accuracy across all conditions and transcription factors would be a major breakthrough with wide-ranging implications for the field.

## 4 Methods

*Data sources*. We use the following TF- or cell type-specific data to train our models:

1. Ground truth TF ChIP-seq labels (DREAM challenge) (ENCODE-DREAM *in vivo* Transcription Factor Binding Site Prediction Challenge n.d)
2. ATAC-seq data from each cell type (ENCODE) (Moore *et al*. 2020).
3. RNA-seq data from each cell type (ENCODE) (Moore *et al*. 2020).
4. Precomputed enhancer activities for 80 ENCODE cell lines and tissues (López Ruiz de Vargas 2021) (uses H3K27ac, H3K4me and H3K4me3 ChIP-seq data).
5. TF motifs from TRANSFAC (Matys *et al*. 2006) version 2022, 1 motif per TF.

The set of ENCODE experimental data used in this work are described in Supplementary Table 2.

Additionally, we use the following data strictly as annotation, given that they do not vary according to the TF or cell type in question:

1. ENCODE cCREs (Moore *et al*. 2020), which defines our set of genomic regions.
2. The hg38 human reference genome.
3. Average ReMap signal track (Hammal *et al*. 2022).
4. 30 primates phastCons (Siepel *et al*. 2005) scores from the UCSC Genome Browser (Hinrichs *et al*. 2006; Nassar *et al*. 2023).

*Feature overview*. We extracted various genomic, cell type, and TF-specific features that collectively capture diverse aspects of regulatory biology. As a genomic feature, we calculated the mean ReMap score (Hammal *et al*. 2022), across all available human TF ChIP-seq experiments, providing a global baseline estimate of TF binding probability. We also employed DNA language model embeddings (Dalla-Torre *et al*. 2025) to obtain high-level sequence representations. Motif-based features include both the maximum TFBS score (the strongest predicted site in the enhancer) and the total affinity of the TF to the sequence, which captures cumulative contributions of weaker binding sites. We further accounted for cell type-specific cooperativity of TF binding sites, drawing on the coTRaCTE framework (van Bömmel *et al*. 2018), to reflect how TFs might cooperatively bind to regulatory regions in a cell type-specific manner. Cell type features include measurements of DNA accessibility (ATAC-seq) and enhancer probability, the latter being precomputed from histone modification ChIP-seq data. Finally, cell-and-TF features include TF expression (from the RNA-seq signal, z-normalized), TF footprinting estimates of TF binding and TF activity (z-normalized) inferred via a regularized linear regression model that predicts enhancer probabilities. The entire set of features are described in Supplementary Table 1.

*Data processing and feature extraction*. We selected a set of approximately 1.5 million genomic regions from the hg38 reference genome, consisting of about 1 million ENCODE cCREs, 200 000 ReMap peaks that do not overlap with the cCREs to enhance the number of potentially bound examples, and 30 000 random genomic regions that do not overlap with either, to enhance the number of unbound examples.

*Ground truth labels for regions*. For each selected region, we determined the ground truth TF ChIP-seq label from the DREAM label data. We excluded the H1-hESC cell line due to the lack of suitable ATAC-seq data, which also resulted in excluding NANOG (only available for H1). Coordinates of each region were lifted from hg38 to hg19 using LiftOver (UCSC) (Hinrichs *et al*. 2006), and then intersected with the DREAM labels using the bedtools intersect tool, such that at least 50% of the bin was intersecting with the region. Since DREAM bins are shorter and also overlapping with each other, this yields 4 to 10 overlapping bins for each region, whose labels need to be summarized into a single label. The bin labels were aggregated with the following rules: if at least 40% of overlapping bins are labeled “bound” (B), then the region is labeled as B. If at least 20% is B and 30% is “ambiguous” (A), then it is labeled as B. If there is at least one B, but less than 20% A, it is labeled A. If there is at least 20% A but no B, it is labeled A. Else, it is labeled “unbound” U. For the training and evaluation of models, only regions labeled as B were considered as positive examples. In the leaderboard set, all regions from chromosome 8 were removed, because all bins from this chromosome were labeled as “ambiguous” in the ENCODE-DREAM challenge files. This process yields, on average, among 230k test genomic regions, 4700 bound (2%) and 11k (5%) ambiguous sites in the final round dataset.

*Motif affinity and TFBS analysis*. MOODS (Korhonen *et al*. 2009) was used to predict TFBS in the regions for each TF in the dataset, yielding the list of all TFBS with a score higher than zero. From this list, the TFBS with the highest score was selected in each region as its maximum PWM score. If a region does not have any TFBS, its maximum score is set to zero. In order to calculate the total affinity of a sequence to a TF, we used the TRAP (Roider *et al*. 2007) tool. TRAP calculates p-values of the hypothesis that the total affinity of a TF to a given sequence is higher than the affinity to a set of control sequences. As control, we used a set of randomly selected 30K ENCODE-ELS enhancer regions. For the model, −log(0.0001+p) was used as the final score, instead of the raw p-value.

*TF footprinting*. TOBIAS (Bentsen *et al*. 2020) version 0.16.1 was used to get TF footprinting signals and bound TF predictions. We used ATACorrect with the ATAC-seq BAM files, the hg38 human reference genome, and IDR thresholded ATAC-seq peaks from ENCODE. Afterwards, the ScoreBigwig and the BINDetect tools were used, the latter with a threshold of 0.001 bound *P*-value.

*RNA-seq expression*. Bulk RNA-seq data from ENCODE was downloaded as gene level TPM quantifications, and for each cell type or tissue, mean TPM was calculated across all available replicates. For the model, log(1+TPM) was used, and data was normalized with z-scores across all examples.

*CRUP enhancer probabilities*. We incorporated enhancer state information from CRUP (Ramisch *et al*. 2019) predictions for 80 ENCODE cell lines and tissues [as precomputed by López Ruiz de Vargas (2021)].

For ATAC-seq signals (fold change over control), TOBIAS TF footprinting, ReMap and phastCons scores, for which we have data in .bw format, bwtool (Pohl and Beato 2014) summary was used to get min, mean and max values within our genomic regions. ATAC-seq data was quantile normalized using the qnorm (https://github.com/Maarten-vd-Sande/qnorm) python package. These multi-level features collectively characterize the regulatory landscape of each region and drive our modeling of TF binding across different cell types.

*Calculating TF activity*. TF activity is inspired by the tool ISMARA (Balwierz *et al*. 2014) and it is defined as the contribution of increased sequence-TF affinity to enhancer activity. It can also be called “TF contribution to enhancer activity”. We calculated TF activity by solving for W in the formula A×W=C, where $A\in R^{N_{enh}\times N_{TF}}$ is the affinity matrix, $C\in R^{N_{enh}\times N_{tissues}}$ is the enhancer activity matrix, $N_{\mathrm{enh}}$ is the number of enhancers, $N_{TF}$ is the number of TFs, and $N_{\mathrm{tissues}}$ is the number of tissues. The matrix A is calculated by scanning enhancer sequences using MOODS with the PWMs of each TF from the TRANSFAC recommended motifs. Then, for each enhancer and TF, the score of the best TFBS in the sequence is selected as the sequence affinity to the TF motif. In cases where there are no TFBS detected in the sequence, the affinity is set to zero. The matrix C is the precomputed CRUP enhancer scores from Ref. 28. The TF activity matrix $W\in R^{N_{TF}\times N_{tissues}}$ is learned through linear regression using the scikit-learn package. Afterwards, TF activities are scaled by a factor of 10000 for easier readability, and scores were z-normalized across all examples.

*Calculating cooperating TFs*. Inspired by the coTRaCTE (van Bömmel *et al*. 2018) framework, we aim to identify TFs that bind cooperatively in a cell type specific manner. We set two integer parameters, *n* = 5000 and *k* = 1000 that control the number of selected enhancers and bound enhancers. First, a list of ubiquitously accessible regions is created, by selecting all enhancers with a mean signal of at least s in all cell types in the training set. The value of s is set such that exactly n enhancers meet this criterion. Denote this set of ubiquitous enhancers by


u={e|mean(e)≥s},  |u|=n.

Next, we generate a list of cell type specific enhancers for each cell type c, by filtering out enhancers with low mean accessibility, then ranking them by their relative deviation from the global mean, and selecting the top n enhancers. Denote this set by $S_{c}$.

Using the TRAP tool, we scan each enhancer $e\in U\cup S_{c}$ for the total binding affinity of every transcription factor τ. We then label the top k enhancers with the highest affinity as bound by the transcription factor τ. We denote by $B_{\tau }$ the set of the top k bound enhancers for τ. Next, we split the total of 2n regions back into the ubiquitous set U and the cell type–specific set $S_{c}$. For a pair of TFs $\left(\tau_{1},\tau_{2}\right)$ we measure cooperative binding by constructing 2-by-2 contingency tables. In $S_{c}$ we count the number of enhancers bound by both $\tau_{1}\;$and $\tau_{2}$ bound only by one or the other, or bound by neither. Denoting these counts by


$$\begin{array}{c} a_{c}=\left|B_{\tau_{1}}\cap B_{\tau_{2}}\cap S_{c}\right|,\\ b_{c}=\left|B_{\tau_{1}}\cap S_{c}\right|-a_{c},\\ c_{c}=\left|B_{\tau_{2}}\cap S_{c}\right|-a_{c},\\ d_{c}=\left|S_{c}\right|-\left(a_{c}+b_{c}+c_{c}\right), \end{array}$$

we apply Fisher’s exact test to obtain a *P*-value, p_cell, that assesses whether $\tau_{1}$and $\tau_{2}$ bind independently in $S_{c}$. We repeat the same procedure in U to get p_ubiq. The cooperation score L is then given by L = log(p_cell/p_ubiq). If L > 2, we say $\tau_{1}\;$and $\tau_{2}$ cooperate in cell type c. To ensure adequate statistical support, we only consider pairs that are co-bound in at least 10 cell type–specific enhancers ($a_{c}\;\geq 10$). Finally, for each TF τ, we select its four strongest partners $\tau^{\prime }$ (those with the highest L) and then compute, for each $\tau^{\prime }\;$across all training enhancers, both the total number of binding sites and the maximum binding-site score. These values, two per co-operating TF, are added to the feature set as eight new features, capturing pairwise binding preferences in a cell type–aware manner.

*Model training*. We implemented a multi-stage model training process using XGBoost (Chen and Guestrin 2016), using gradient-boosted decision trees to predict TF binding probabilities. First, the general model is trained on the complete training set, where each training enhancer is repeated as many times as there are different TF-cell type pairs. Predictions from this general model were included as an additional feature in the subsequent training stage of TF-tuned models. These are trained specifically on the subset of the training data with ChIP-seq data from a particular TF. Therefore we end up with a different TF-tuned model for each different TF. Further, TF-only and TF+transformer models were trained separately for each TF, using only training data corresponding to that TF.

All models were trained using XGBoost with the gradient boosting tree method. The objective function was set to logistic regression to predict TF binding probabilities. Training parameters were set close to default values, including n_estimators = 500, learning rate = 0.05, subsample = 0.8, column sample = 0.8, tree depth = 6, min_child_weight = 1. Training was performed on an A100 GPU using CUDA cores.

*“Criss-cross” ensembling*. For our ensemble models, in order to make better predictions on previously-unseen cell types, the “criss-cross training” idea from Li *et al*. was used with some modifications (Li *et al*. 2019). Given a TF, we split the available training tissue types randomly into two groups called A and B. Likewise we split the training chromosomes randomly into two groups called 1 and 2. Then, we train 4 models for each TF: the first model is trained on tissue types A and chromosome group 1, while being validated on tissues B and chromosomes 2. The second model is trained on A2, validated on B1 etc. Each model was trained until its performance on the respective validation set failed to improve for 50 boosting rounds. The final prediction is computed as the mean of the four models’ outputs.

## Supplementary Material

vbag123_Supplementary_Data

## Data Availability

The data underlying this article are available at https://zenodo.org/records/19457863. The set of ENCODE experiment IDs used in this work are described in Supplementary Table 2.

## Code availability

The code used to extract features, train models and reproduce the figures is available at https://github.com/ekinda/tfbs_prediction_paper.
