# Supplementary material for: Modeling strategies for in vivo transcription factor binding predictions
Source: Bioinform Adv. 2026 May 5;6(1):vbag123. doi: 10.1093/bioadv/vbag123 (PMC13197119; doi:10.1093/bioadv/vbag123)
Supplement: vbag123_Supplementary_Data [file vbag123_supplementary_data.zip › sup_table1.pdf]

Supplementary Table 1. List of handcrafted features used in the models

Number	Name	Depends on		
		Region	TF	Cell type
1	Maximum TFBS score	x	x	
2	Total TFBS affinity	x	x	
3	Average phastCons	x		
4	Average ReMap	x		
5	Average CRUP score	x		x
6	Mean CRUP score of region across all cell types	x		
7	Difference between (5) and (6)	x		x
8	TF expression as average TPM in cell line		x	x
9	TF activity		x	x
10	TF activity - CRUP correlation p-value	x	x	x
11	TF activity - CRUP correlation coefficient	x	x	x
12	Minimum ATAC signal from region	x		x
13	Mean ATAC signal from region	x		x
14	Maximum ATAC signal from region	x		x
15	Delta (12) (compared to mean across all samples)	x		x
16	Delta (13)	x		x
17	Delta (14)	x		x

18	Average of (13) across all samples	x		
19	Average TOBIAS score in region	x		x
20	Average of (19) across all samples	x		
21	Difference between (20) and (19)	x		x
22	Number of “bound” TFBS calls from TOBIAS	x	x	x
23	Maximum TFBS score in sequence, using Cotracte TF #1	x	x	x
24	Maximum TFBS score, Cotracte TF #2	x	x	x
25	Maximum TFBS score, Cotracte TF #3	x	x	x
26	Maximum TFBS score, Cotracte TF #4	x	x	x
27	Number of TFBS in region, using Cotracte TF #1	x	x	x
28	Number of TFBS in region, using Cotracte TF #2	x	x	x
29	Number of TFBS in region, using Cotracte TF #3	x	x	x
30	Number of TFBS in region, using Cotracte TF #4	x	x	x
31	Prediction from the general model (only in TF-tuned)	x	x	x
32-1055	Nucleotide Transformer embeddings (only in transformer)	x		
