# Supplementary material for: Modeling strategies for in vivo transcription factor binding predictions
Source: Bioinform Adv. 2026 May 5;6(1):vbag123. doi: 10.1093/bioadv/vbag123 (PMC13197119; doi:10.1093/bioadv/vbag123)
Supplement: vbag123_Supplementary_Data [file vbag123_supplementary_data.zip › supplementary_figures.pdf]

Modeling strategies for in vivo transcription factor binding predictions

Ekin Deniz Aksu, Martin Vingron
Max Planck Institute for Molecular Genetics
5 April 2026

Supplementary Figures

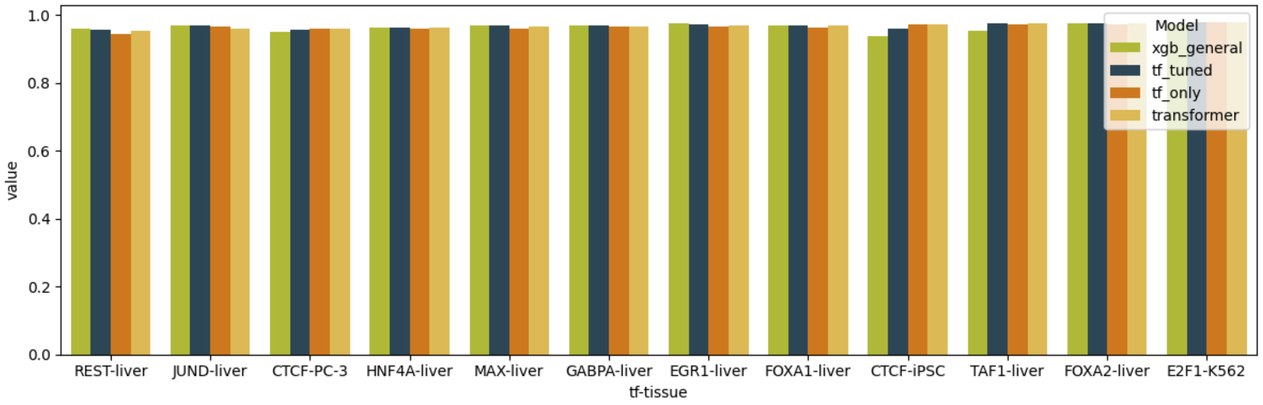

Figure S1: AUROC in the test set

Area under the reciever-operating characteristic curve (AUROC) in the test set. Due to the unbalanced nature of the dataset, AUROC is not a suitable evaluation metric.

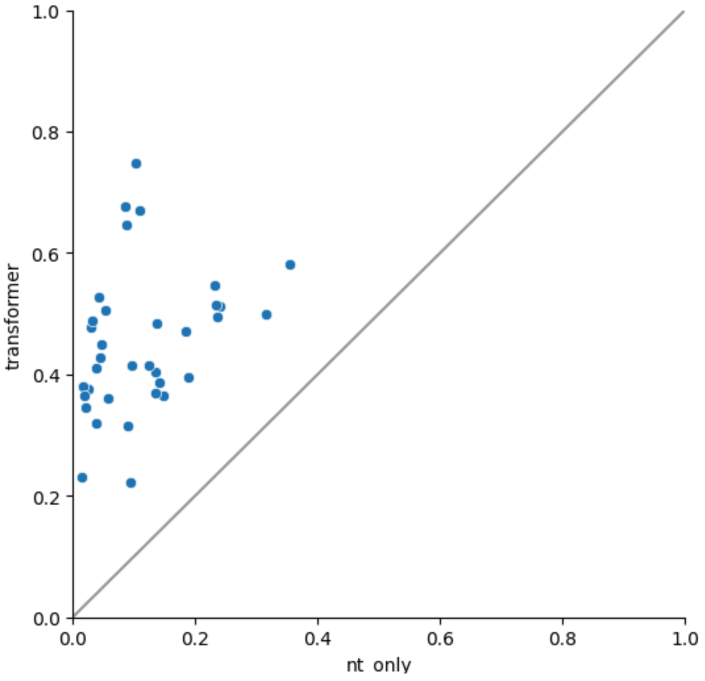

Figure S2: Transformer model vs embedding-only model

AUPR comparison in the final round and leaderboard sets. The y-axis shows AUPR of the TF+transformer model, and the x-axis shows AUPR of models with features constrained to only 1024 Nucleotide Transformer embeddings. Our TF+transformer model consistently outperforms models trained only using Nucleotide Transformer embeddings.

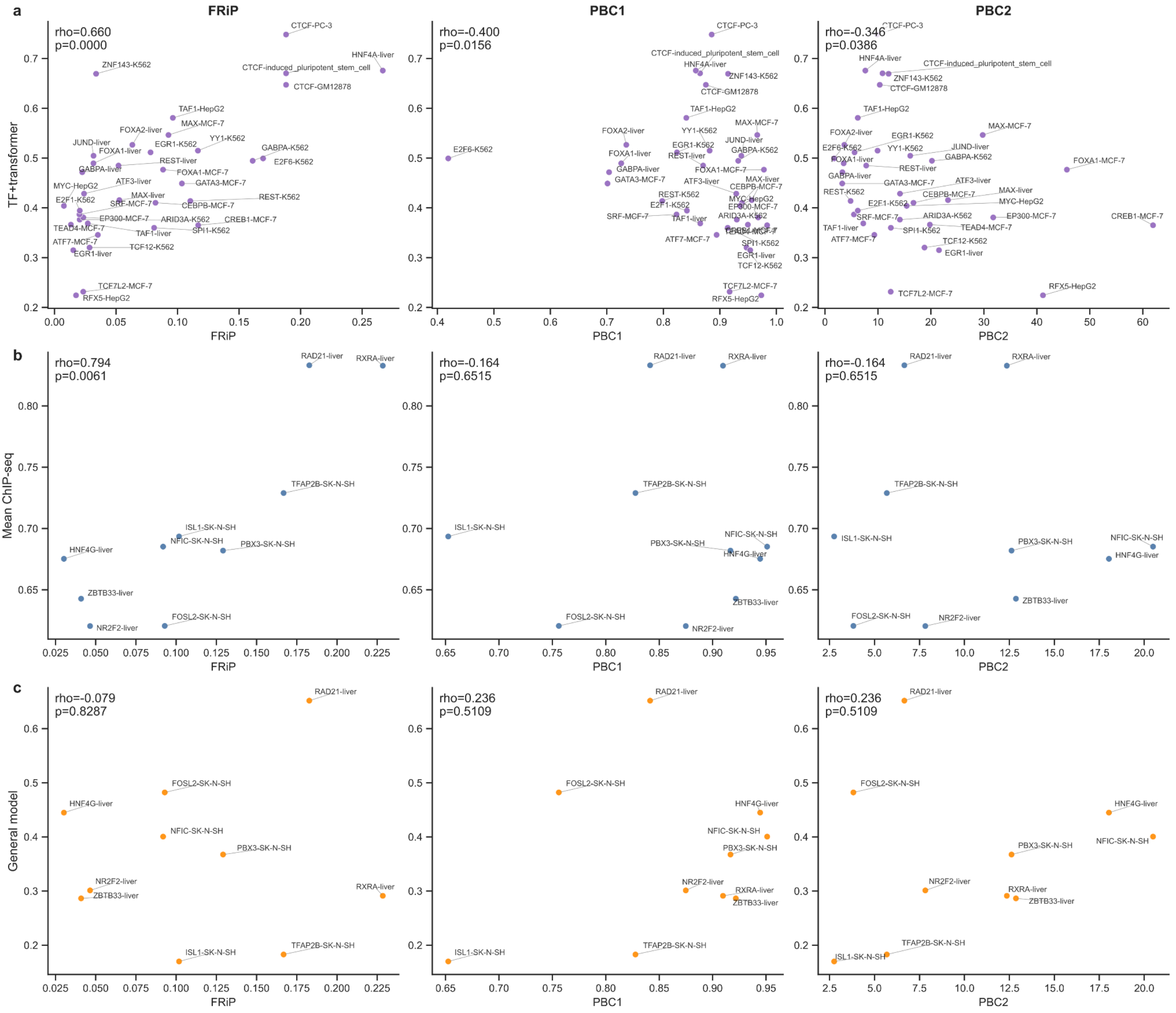

Figure S3: ChIP-seq data quality metrics

(a) shows the relationship between the AUPR score of the TF+transformer model in the final round and leaderboard datasets versus FRiP, PBC1 and PBC1 scores. Spearman correlation coefficients and p-values are written inside the plots. (b) shows the same relationships in the “new TFs” dataset using the mean ChIP-seq score and (c) shows the same relationships in the “new TFs” dataset using the general model prediction score.

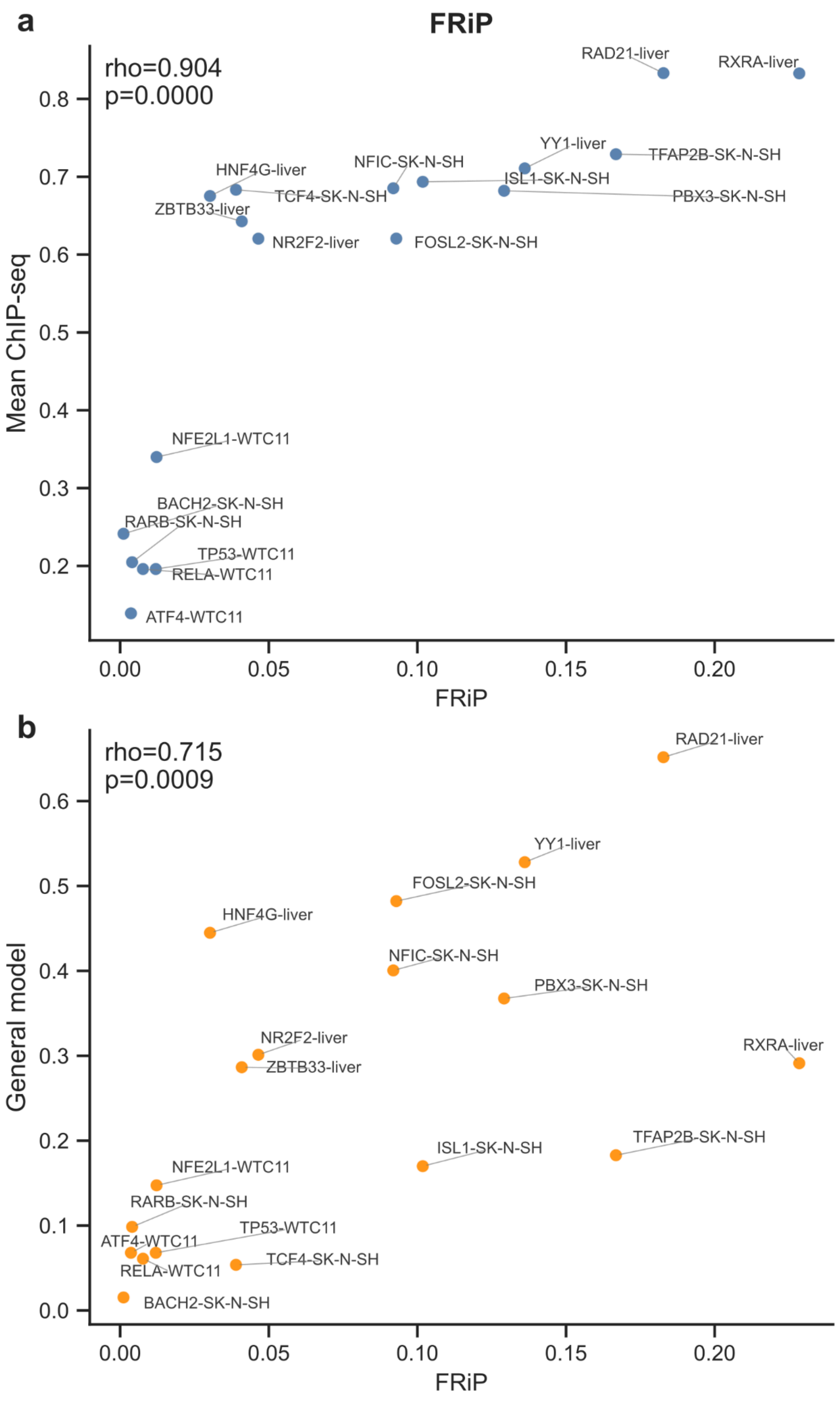

Figure S4: Relationship between FRiP and predictions in the expanded dataset

Data from the expanded “new TF” dataset is shown, including new ChIP-seq experiments with low FRiP scores.

a

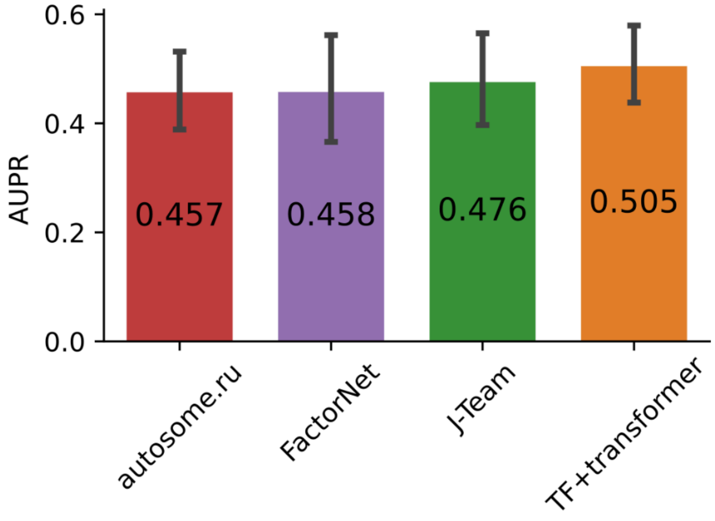

b

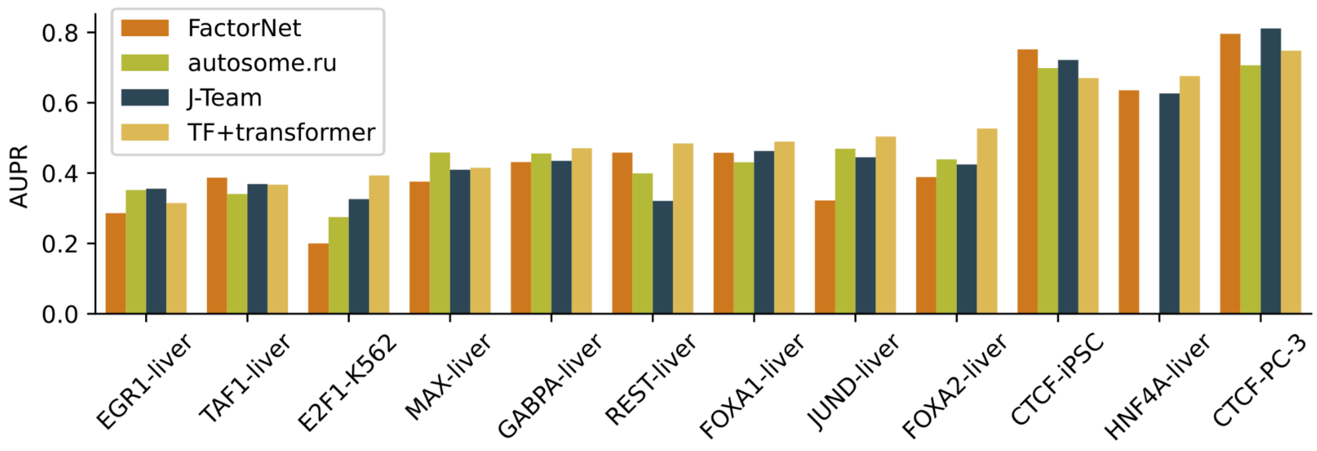

Figure S5: Comparison to DREAM challenge contestants

(a) Mean AUPR scores in the test set of the ENCODE-DREAM challenge. The bars show the mean performance across all TFs. Note that submitted predictions for the other models were used, using the original DNase-seq training data.

(b) Performance across different TFs for the same models.

a

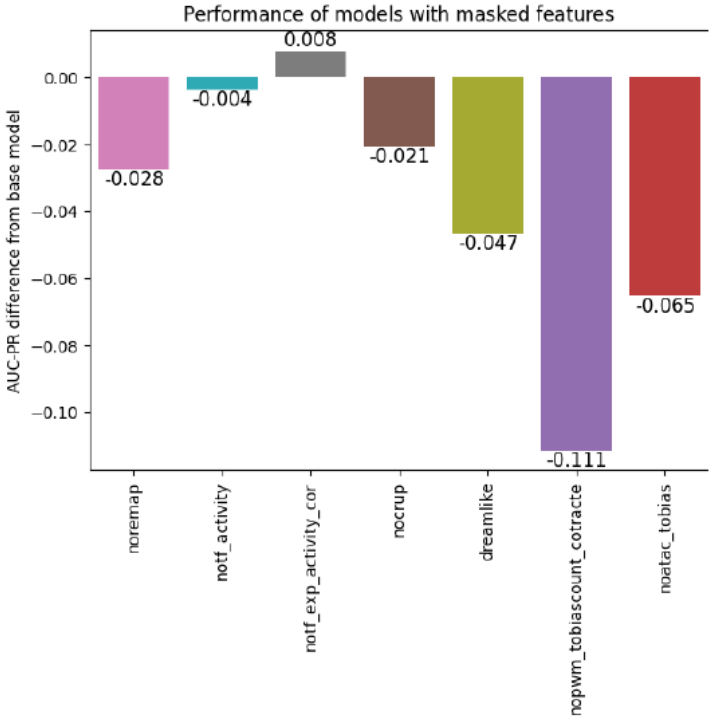

b

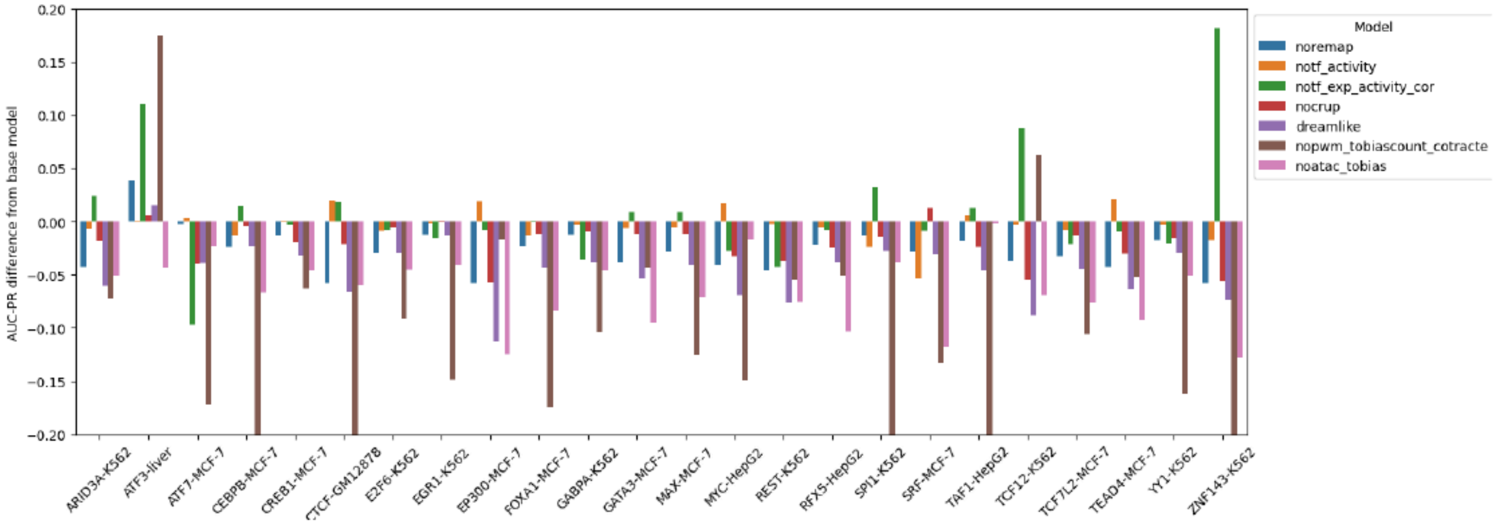

Figure S6: Feature contributions in the leaderboard set

(a) Delta AUPR compared to the full model after masking feature sets, in the leaderboard set

(b) Feature contributions across different samples in the leaderboard set.

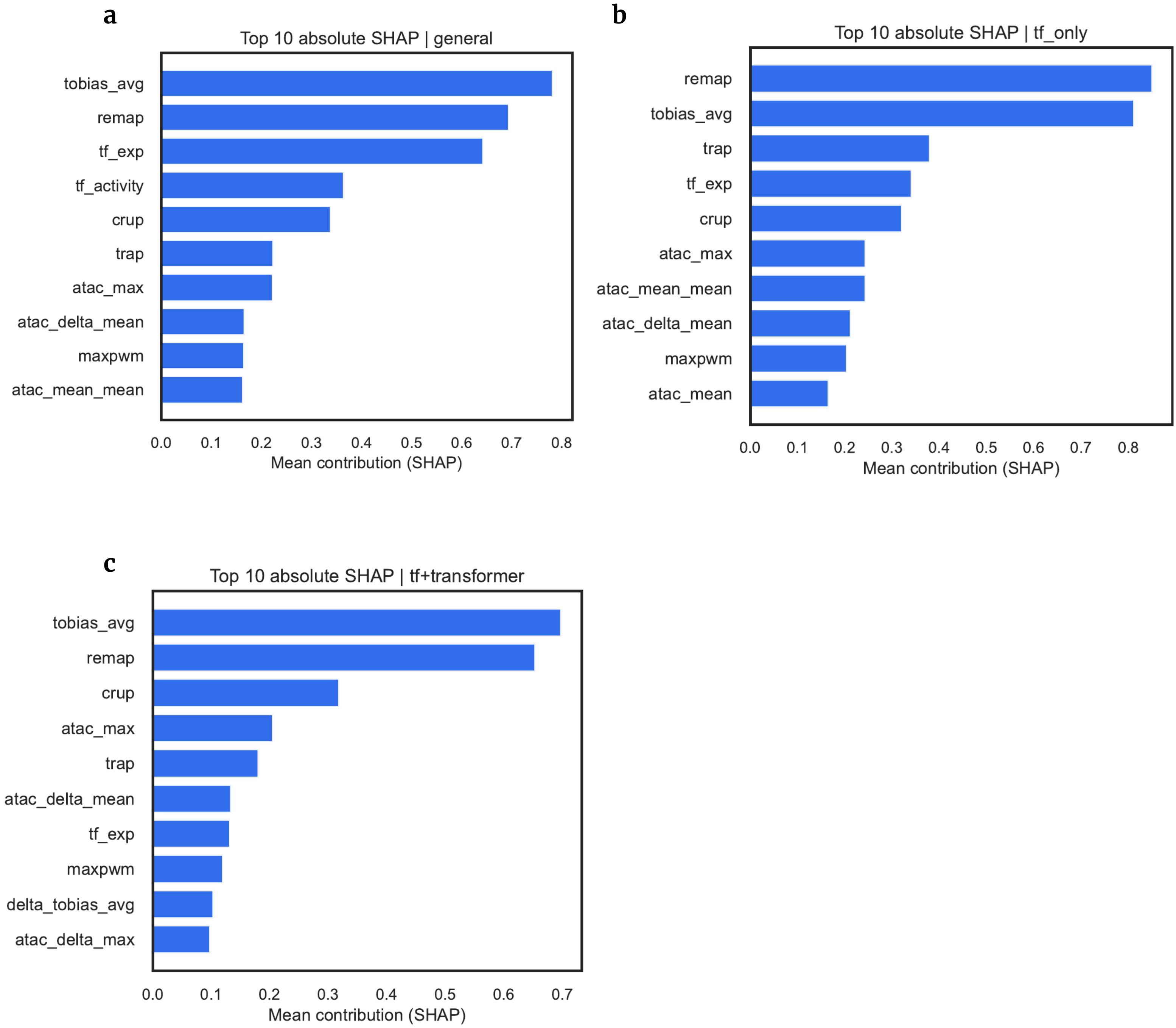

Figure S7: Average absolute SHAP values for different models

Figure shows top 10 features ranked by absolute SHAP value in the (a) general, (b) TF-only, and (c) TF+Transformer models.

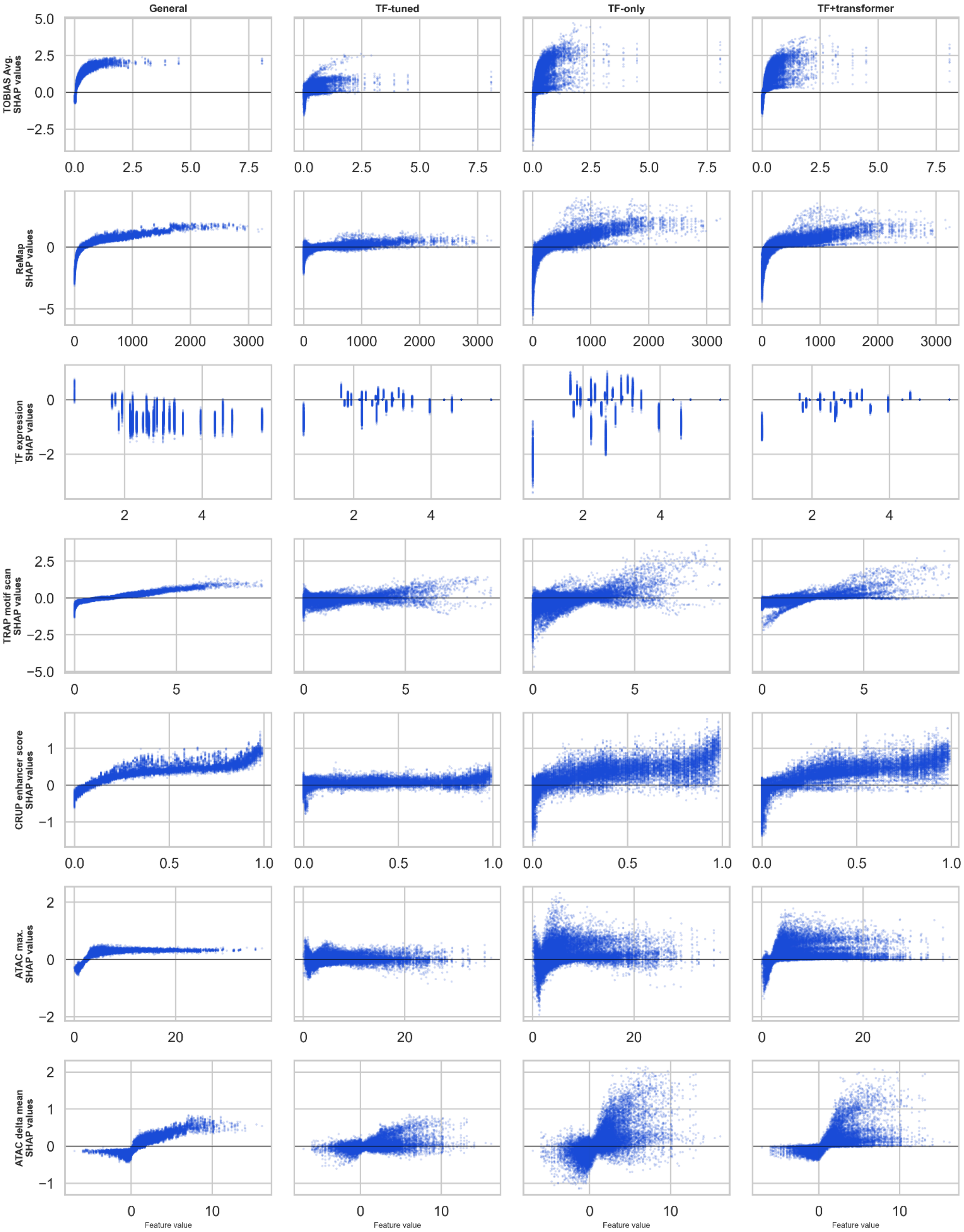

Figure S8: SHAP dependence plots

Figure shows relationships between feature values (x-axis) and SHAP values (y-axis). Each row corresponds to a different feature, and each column corresponds to a different model.

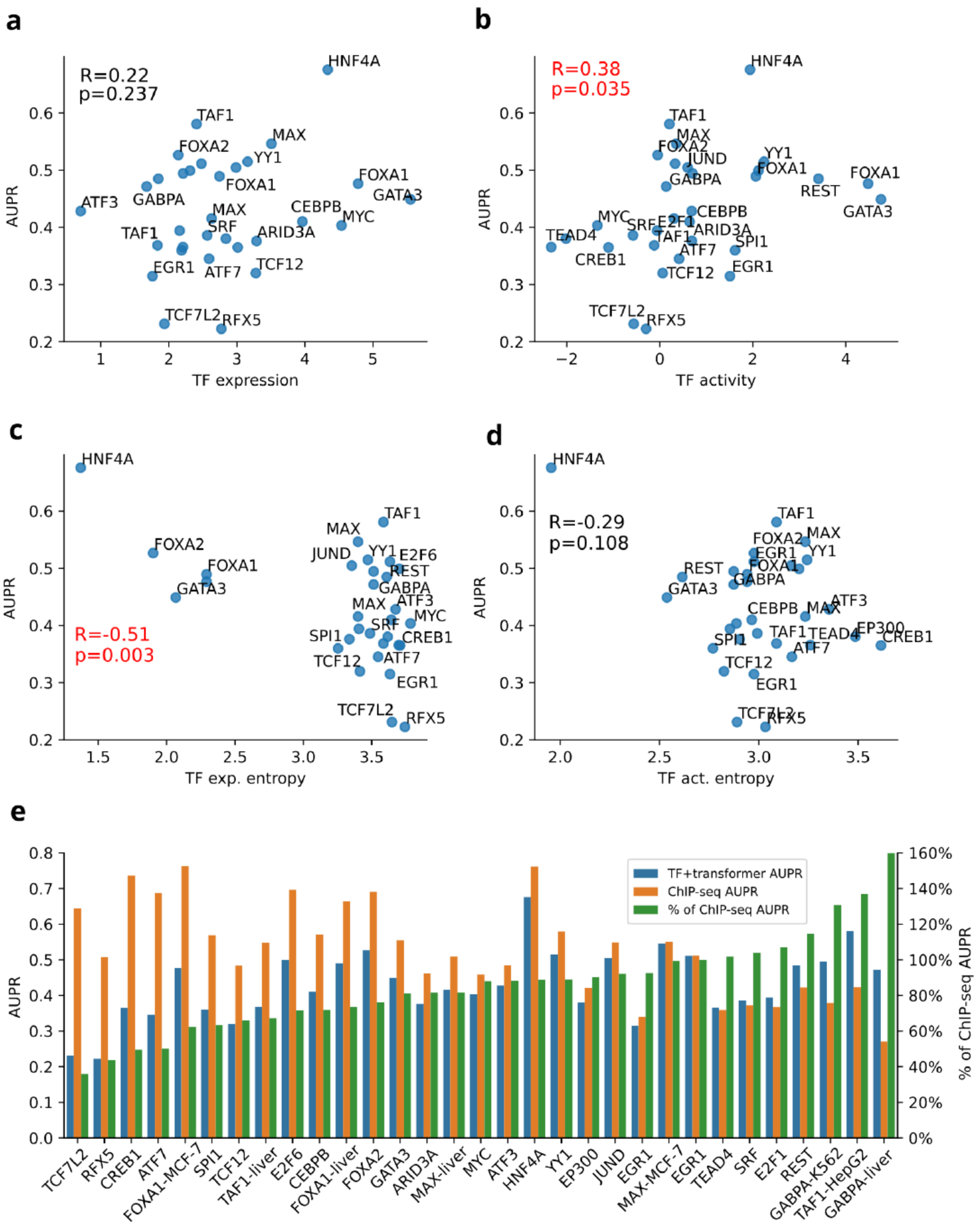

Figure S9: Determinants of model prediction performance

(a-d) Scatterplots illustrating the relationship between model performance and TF expression, TF activity, TF expression entropy and TF activity entropy. Pearson correlation coefficients (R) and p-values are shown, with significant correlations marked in red. (e) Comparison of model performance against predictions based on the average ChIP-seq signal. Green bars represent the ratio of model performance to ChIP-seq performance.
